# Immunohistochemical analysis of chromatin remodeler DAXX in high grade urothelial carcinoma

**DOI:** 10.1186/1746-1596-8-111

**Published:** 2013-07-02

**Authors:** Antonio Zizzi, Maria Alessandra Montironi, Roberta Mazzucchelli, Marina Scarpelli, Antonio Lopez-Beltran, Liang Cheng, Nicola Paone, Paolo Castellini, Rodolfo Montironi

**Affiliations:** 1 Section of Pathological Anatomy, Polytechnic University of the Marche Region, School of Medicine, United Hospitals, Via Conca 71, 60126, Torrette Ancona, Italy; 2 Department of Industrial Engineering and Mathematical Sciences (DIISM), Polytechnic University of the Marche Region, Ancona, Italy; 3 Department of Surgery, Cordoba University Medical School, Cordoba, Spain; 4 Department of Pathology and Laboratory Medicine, Indiana University School of Medicine, Indianapolis, IN USA

**Keywords:** Urothelium, Urothelial carcinoma, Chromatin remodeler DAXX, Biological regulator

## Abstract

**Background/Aims:**

The chromatin remodeler DAXX, a predominantly nuclear protein, regulates the status of chromatin organization. The aim of this exploratory immunohistochemical study was to evaluate DAXX protein expression in high grade invasive urothelial carcinoma (UC) of the bladder as a biological regulator of aggressiveness.

**Methods:**

Quantitative analysis was made on DAXX immunostained nuclei in tissue sections from 5 cases of bladder normal urothelium (NU) and 5 cases of bladder pT1 UC. Carcinoma in situ (CIS) and high grade papillary carcinoma (HGPCa) were identified in 2 out of 5 UC cases.

**Results:**

The nuclei in UC show an open configuration of the chromatin composed of granules varying in size and distribution and a mean nuclear area 1.7 times greater than that in NU (UC: mean and SD 24.4 ± 11.4 square microns; NU: 14.8 6.5 square microns. The differences are statistically significant). 70% of the NU nuclei are immunostained, whereas 90% of UC nuclei are positive. The mean gray level value in UC, related to the intensity of nuclear immunostaining, is lower than in NU by a factor of 0.94 (UC: mean and SD 100 ± 15; NU: 106 ± 15. The differences are statistically significant). In particular, the value in the nuclei adjacent to the stroma in UC is slightly lower than in the intermediate cell layers by factor of 0.98, whereas in NU it is slightly greater by a factor 1.02 and 1.04 compared to the intermediate and superficial cell layers. The values in CIS and HGPCa are similar to those in UC.

**Conclusions:**

The quantitative immunohistochemical analysis shows an altered protein expression of chromatin remodeler DAXX in UC and in its preinvasive phases, when compared to NU. DAXX evaluation, if associated with markers related to global DNA methylation and histone acetylation, could be used in clinical practice as a marker of aggressiveness.

**Virtual slides:**

The virtual slides for this article can be found here:

http://www.diagnosticpathology.diagnomx.eu/vs/1398457297102379

## Background

Urinary bladder cancer is the fifth most common cancer in the western world. The relative 5-year survival rate of ≈ 70% has not changed the last few decades [1]. The clinical presentation of the disease is heterogeneous, ranging from tumors with low malignant potential to highly malignant muscle infiltrating tumors [2]. Despite refined histology-based classification systems, it is difficult to predict the individual prognosis and response to therapy. For instance, a third of patients with T1 tumors remain recurrence-free after BCG treatment, while a third die from the disease. Therefore clinical biomarkers identifying aggressive tumors are clearly desirable [3].

Gene-expression signatures associated with different clinical variables such as cancer progression have been proposed. One major drawback is that gene-expression analyses require access to frozen tissue for extraction of high-quality DNA and RNA. However, in clinical routine, mainly formalin fixed paraffin-embedded tissue samples have been preserved and archived. To retrieve information at protein level from these invaluable materials, with long and extensive patient follow-up data, immunohistochemistry is an ideal approach to identify potential new biomarkers [3].

Several biological regulators of aggressiveness have been suggested as biomarkers in bladder cancer, including p53, p21, Rb, FGFR3 and survivin [4-7]. However, results are often inconclusive and the clinical impact remains poor. Previous immunohistochemical investigations made by our group showed the status of chromatin organization, evaluated with global DNA methylation and histone acetylation analysis, to have a potential prognostic value in UC [8,9].

The chromatin remodeler DAXX, a predominantly nuclear protein, often localized within subnuclear compartments called PML (promyelocytic leukemia protein) oncogenic domains, regulates the status of chromatin organization [10-13]. Recent studies on formalin fixed paraffin-embedded tissue samples have linked chromatin remodeler DAXX expression with tumor aggressiveness in cancer patients [14-18].

The aim of this exploratory study was to investigate quantitatively the immunohistochemical expression of chromatin remodeler DAXX in T1 high-grade UC of the bladder as a potential biological regulator of tumor aggressiveness.

## Methods

### Patients and tissue samples

The procedure for this research project conforms to the provisions of the Declaration of Helsinki. The study included 5 cases of bladder NU obtained from patients with benign prostatic hyperplasia and no history of bladder and prostate cancer and 5 cases of bladder pT1 UC. The material was retrieved from the Pathology Services associated with Polytechnic University of the Marche Region-United Hospitals. All the cases had been fixed in 4% buffered formaldehyde for approximately 24 hours before processing. The hematoxylin- and eosin-stained sections were retrieved from the archives and reviewed by one of our team (RM). The original diagnosis of NU and of invasive UC was confirmed in all the cases. A small component of CIS and of HGPCa was identified in 2 out of 5 UC cases.

### Immunohistochemistry

Five-micron thick sections were cut from the paraffin blocks, dewaxed in xylene and rehydrated through a graded series of ethanol. Antigen retrieval was done by microwave treatment for 20 min at 98°C using 0.01 M Citric Acid buffer pH 6.0. Endogenous peroxidase activity was quenched by incubating the sections in 3% hydrogen peroxide for 10 min at room temperature. Non-specific binding sites were blocked through pre-incubation with 5% normal goat serum in PBS for 10 min at room temperature. DAXX immunostaining was performed using a polyclonal antibody (1:100, Sigma). Antigen-antibody complex was subsequently visualized using the Envision™ Detection System kit peroxidase/DAB (DAKO, Glustrop, Denmark) and counterstained with hematoxylin. Negative controls were used for the tested antibodies; the primary antibody was replaced by either mouse or rabbit non-immune serum, as appropriate.

### Evaluation of immunohistochemistry

Several images representative of NU, CIS, HGPCa and UC were recorded with a Nikon digital camera mounted on a Nikon Eclipse E800 microscope at the objective magnification of 20×. The nuclear area (unit of measurement: square microns; calibration was based on the measurement of nuclei with known diameter) and the intensity of nuclear staining (unit of measurement: gray levels) were analyzed with the LabVIEW software (National Instruments, Austin, Texas). For the NU cases and in the two cases of CIS and HGPCa associated with UC the nuclei were separately evaluated in the following three compartments: cell layer adjacent to the stroma, i.e., basal cells; superficial or luminal cell layer; and intermediate cells, i.e., those between the basal cells and superficial cells. Since superficial cells are not present in UC, the nuclei were evaluated in the cell layer adjacent to the stroma, the nuclei in all the other cell layers being considered equivalent to the intermediate nuclei of NU. At least 40 nuclei per location were measured in each case, for a total of 1300 nuclei. For each nucleus the mean nuclear gray value was calculated from the gray value of the individual picture elements in the green color plane. The nuclei were pooled together and the mean and standard deviation of the nuclei in each group and for each location was calculated. This was due to the fact that the number of cases analyzed in each group was reactively small. However, an attempt made to calculate the mean and standard deviation per each group based on individual cases did not shown substantial differences with the figures obtained from the pooled nuclei.

Since the aim of this study was the feasibility of quantifying DAXX immunostaining as a potential biological regulator of aggressiveness, statistical analysis was applied to a limited extent only (Student’s T test; significance at p< 0.05) and not throughout all the steps the study.

## Results

### Evaluations on hematoxylin- and eosin-stained sections

The nuclei of NU are round to oval. Visually they are almost two times the size of the nuclei of the small lymphocytes in the subepithelial connective tissue. The chromatin is slightly less condensed compared to the hyperchromatic nuclei of the lymphocytes. The nucleoli are not visible. Mitotic figures are absent. The nuclei in UC, at least three times larger than the nuclei of the lymphocytes, show an open configuration of the chromatin composed of granules varying in size and distribution. The nucleoli are prominent and visible in the majority of nuclei. The CIS and HGPCa nuclei, including their chromatin distribution, are morphologically indistinguishable from those in UC. Mitotic figures are present in all UC cases, including in the noninvasive CIS and HGPCa components. There is neither acute nor chronic inflammation other than scattered lymphocytes randomly distributed in the subepithelial connective tissue.

### Qualitative and quantitative valuations on DAXX immunostained slides

#### Invasive urothelial carcinoma vs. normal urothelium

Karyometric analysis further defines the nuclear size in addition to that based on the subjective evaluation on hematoxylin- and eosin-stained slides. The nuclear size in UC is 1.7 times greater than that in NU (UC: mean and SD 24.4 ± 11.4 square microns; NU: 14.8 ± 6.5 square microns. The differences are statistically significant). The nuclei in the cells adjacent to the stroma in UC and in the basal cells in NU are slightly smaller than in the intermediate cells by a factor of 0.96 and 0.86 in the UC and NU cases, respectively, the difference being not statistically significant. The mean nuclear area of the nuclei in the superficial cell layer in NU is identical to the nuclei in the intermediate cell layers. This means that the nuclei increase in size when they migrate from basal or adjacent to the stroma position to the intermediate location, but the size does not change in NU when the nuclei reach the surface.

**Figure 1:**
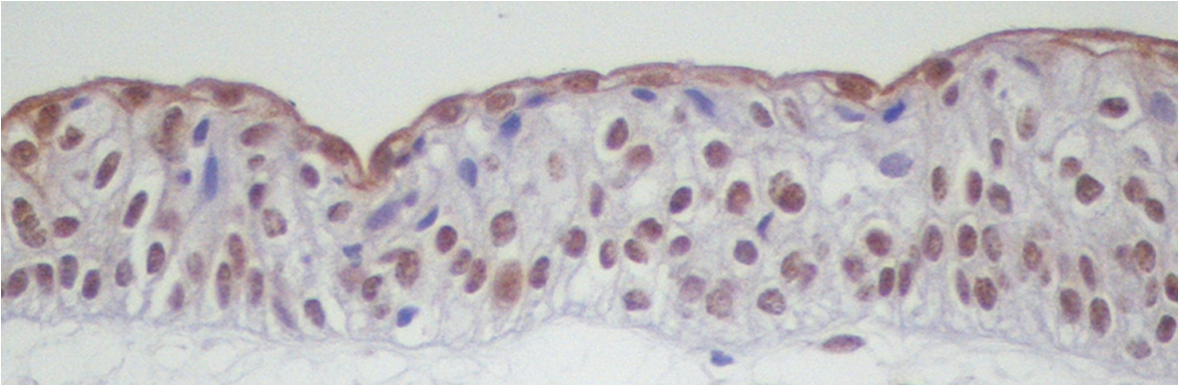
**DAXX immunostaining in normal urothelium.** Nuclear staining is present in basal, intermediate and superficial position. Not all the nuclei are stained. Note that the intensity of immunostaining is not homogeneous throughout the cell layers. The superficial or umbrella cells show some stating in the cytoplasm in addition to that of the nuclei. (Original microscope objective magnification 20×).

**Figure 2:**
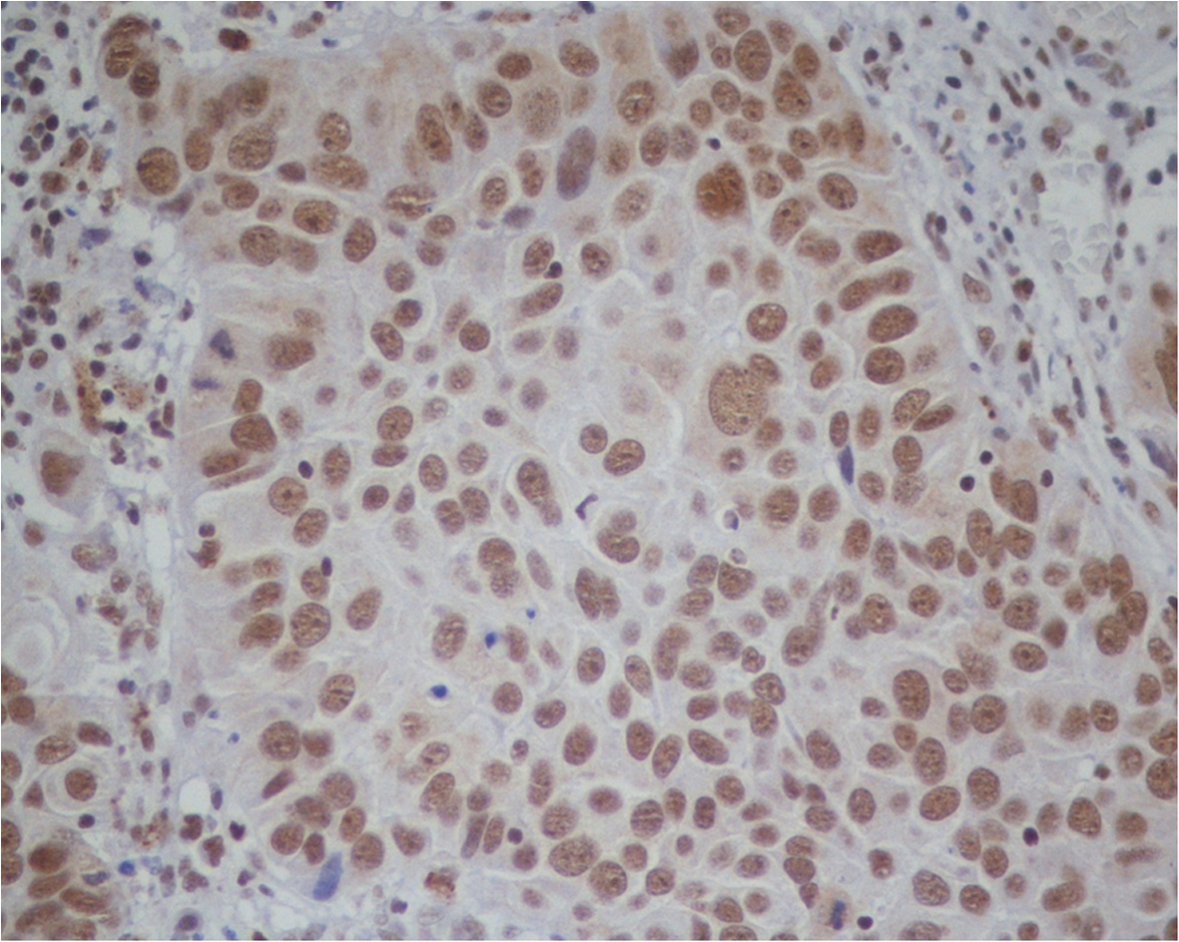
**DAXX immunostaining in urothelial carcinoma.** The majority of nuclei are stained at all cell levels. Nuclear staining is not homogenous, some nuclei being darker than others. No cytoplasmic staining is present. (Original microscope objective magnification 20×).

The DAXX antibody stains the nuclei in all diagnostic groups and all nuclear locations (Figures 1 and 2) (Table 1). In particular, 70% of the NU nuclei are immunostained, whereas 90% of UC nuclei are positive. The intensity of staining of the positive nuclei ranges from weak-to-moderate to strong. There is no spatial association between strong stained nuclei or negative nuclei and mitoses. Some faint immunostaining is seen in the cytoplasm of scattered epithelial cells, both in NU, mostly in the superficial cell layer (Figure 1), and UC. Nonepithelial cells in the stroma, including smooth muscle cells and endothelial cells, are not stained. Quantitative evaluation was done on the epithelial nuclei only.

**Table 1:** DAXX immunohistochemistry results in normal urothelium and in urothelial carcinoma

	**Mean (±SD) nuclear gray level**
Normal urothelium (NU), global	106±15
NU, superficial epithelial layer	104±15
NU, intermediate cell layers	106±15
NU, basal cell layer	108±14
Urothelial carcinoma (UC), global	100±15
UC, nuclei not adjacent to the stroma	101±15
UC, nuclei adjacent to the stroma	99±16

The mean nuclear gray value in UC is lower than in NU by a factor of 0.94 (UC: mean and SD 100 ± 15; NU: 106 ± 15. The differences are statistically significant). The value in the nuclei adjacent to the stroma in UC is slightly lower than in the intermediate cell layers by factor of 0.98, whereas in NU it is slightly greater by a factor 1.02 and 1.04 compared to the intermediate and superficial cell layers, i.e., the trend of DAXX protein expression changes in the various nuclear locations in NU is different from that in UC, being decreasing in the former and increasing in the latter. This points out that DAXX protein expression in UC is different from that in NU in terms of staining intensity and nuclear location.

DAXX staining is not homogeneous within each individual nucleus. It shows a granular pattern, some granules being darker than others. An attempt was made to further characterize the nuclear staining pattern in UC compared with NU based on the highest gray value per nucleus. Such an approach does not add information behind that derived from the mean nuclear gray value per nucleus (further data not shown).

#### Carcinoma in situ and high grade urothelial papillary carcinoma

There are no differences between CIS and HGPCa concerning nuclear size and mean gray values related to DAXX immunostaining. Compared to NU, the nuclear area is greater by a factor of 1.7, as in UC. The trend of changes in the three nuclear locations is similar to that in NU, i.e., increasing from the basal position to the surface. The mean nuclear gray values in CIS and HGPCa are similar similar to that invasive UC and lower than in NU, however they retain the pattern of changes in the nuclear locations seen in NU, i.e., decreasing from the basal position to the surface.

## Discussion

The current study shows two major findings. One is represented by the significant changes in nuclear size and chromatin organization in UC and its two preinvasive lesions compared with NU. The other is represented by an altered expression of the chromatin remodeler DAXX in UC as well as in CIS and HGPCa, compared with NU. The nuclear size depends on the DNA content as well as on the status of chromatin organization. The latter depends on epigenetic events, such as DNA methylation and histone modifications, and is regulated by the chromatin remodeler DAXX [10-13].

Compared to normal cells, DNA of cancer cells is generally hypomethylated, while promoters of certain genes are hypermethylated, in the context of CpG islands. Such promoter-specific increase in methylation leads to silencing of the affected gene that might have functioned as, for instance, a tumor suppressor. Transcriptional repression by DNA methylation is mediated by a class of methyl DNA binding proteins which, by virtue of recognizing specifically methylated DNA sequences, recruit repressive protein complexes including histone deacetylases to gene promoters (see below) [8,9,19]. The combination of CpG island methylation, proteins that binds to them, and repressive histone modifications generates localized regions of specialized chromatin, which can inhibit transcription. Despite a growing list of genes including tumor suppressors and DNA repair genes that are aberrantly hypermethylated in different cancers, only a limited number of the identified hypermethylated genes have demonstrated any potential utility in clinical decision making. As opposed to single-gene analysis, the integrated information on methylation patterns of multiple genes may reflect the functional status of several cellular pathways [19,20].

To investigate DNA methylation in situ an immunohistochemical approach was adopted by our group using a monoclonal antibody that recognize the presence of a methyl group on the carbon 5 of cytosine. This allowed the analysis of global methylation to be performed on interphase nuclei, on a cell by cell basis by microscopy [20]. Negative nuclei and those with weak-to-moderate intensity were considered unmethylated and hypomethylated, respectively, whereas those strongly stained as hypermethylated. That study showed an altered global DNA methylation pattern in UC compared with NU, the proportion of nuclei with weak-to-moderate intensity in the former being greater than that of strongly stained. This was interpreted as an increased global DNA unmethylation and hypomethylation in UC compared with NU [8,9].

Histones are subject to a variety of post-translational modifications, including acetylation of lysines. Such modifications play fundamental roles in gene regulation and other chromatin-based processes. Histone-modifying enzymes affect histones either locally, through targeted recruitment by sequence specific transcription factors [21], or globally throughout the genome in an untargeted manner, affecting virtually all nucleosomes [22]. Such widespread functions that occur independently of apparent sequence-specific DNA binding proteins are referred to as global histone modifications. Like their targeted effects, the global activity of the histone modifying enzymes can modulate gene activity [22]. Therefore, histones are modified locally and globally through multiple histone-modifying enzymes with different substrate specificities, generating hierarchical patterns of modifications from single promoters to large regions of chromosomes and even single cells.

Since histone modifications occur throughout the genome, any potential change in the activity of the histone modifying enzymes results in changes in specific histone patterns detectable at the level of individual nuclei by immunohistochemistry. While the immunohistochemical approach provides information on global histone acetylation, it does not give information on the genomic, gene–gene differences in distribution of histone modifications. To investigate global histone acetylation in UC an immunohistochemical approach was previously adopted by our group using a polyclonal antibody raised against acetylated H3K9 (i.e., lysine 9 (K9) of histone 3) [8,9]. The study showed an altered pattern of global histone acetylation in UC, the percentages of positive nuclei being higher than in NU.

These two previous investigations pointed out that the chromatin pattern in UC is associated with an increased global DNA unmethylation and hypomethylation and with an altered pattern of global histone acetylation compared with NU [8,9]. The current study, performed on the same series of cases, indicates that such changes in UC are associated with an increased number of DAXX positive nuclei whose intensity of nuclear immunostaining is lower than in NU by a factor of 0.94. Since DAXX is a repressor of gene expression that binds DNA methyl transferases, histone deacetyl transferases and chromatin-modifying proteins, the findings in the current study are of paramount importance to explain the changes in DNA methylation and histone acetylation in UC.

There is only one previous study by Segersten et al. [3] on DAXX immunohistochemistry in bladder neoplasms. The investigation was performed on tissue microarray (TMA) material comprising a range of noninvasive and invasive bladder cancer. The aim was to screen a publicly available immunohistochemistry based web-atlas to identify key proteins that might serve as potential biomarkers. The study did not focus on DAXX, as in our study, but involved a series of proteins. The expression of ALCAM, CN130, DAXX, GAL1, PHF6 and XPA were significantly correlated with tumor grade and stage. ALCAM and GAL1 expression were increased with advanced stage, whilst the others were decreased. In particular, GAL1 expression was higher in poorly differentiated tumors, whilst DSG3 and DAXX protein expression was decreased. Our current study confirms Segersten et al’s findings of a decreased DAXX protein expression in UC and expands our knowledge in this field because it shows that its altered expression correlates with previously observed changes in DNA methylation and histone acetylation.

In conclusion, the quantitative immunohistochemical analysis shows an altered expression of chromatin remodeler DAXX in UC and in its preinvasive phases, when compared with NU. In particular, the evaluation of the DAXX protein expression, if associated with other markers related to global DNA methylation and histone acetylation, could be used in clinical practice as a marker of aggressiveness.

## Competing interests

The authors declare that they have no competing interests.

## Authors’ contributions

AZ, MAM and RM constructed the manuscript. RM, RMa and MS carried out pathologic study. MAM, NP and PC analyzed with the LabVIEW software; AL-B, LC and RM were responsible for clinical data. evaluated clinical data; formed analysis of relation between clinical data and pathologic data; RM designed and constructed manuscript. All authors read and approval the final manuscript.
